# Harmonization of maternal balanced energy-protein supplementation studies for individual participant data (IPD) meta-analyses – finding and creating similarities in variables and data collection

**DOI:** 10.1186/s12884-023-05366-2

**Published:** 2023-02-11

**Authors:** Alison D. Gernand, Kelly Gallagher, Nita Bhandari, Patrick Kolsteren, Anne CC Lee, Yasir Shafiq, Sunita Taneja, James M. Tielsch, Firehiwot Workneh Abate, Estifanos Baye, Yemane Berhane, Ranadip Chowdhury, Trenton Dailey-Chwalibóg, Brenda de Kok, Neeta Dhabhai, Fyezah Jehan, Yunhee Kang, Joanne Katz, Subarna Khatry, Carl Lachat, Sarmila Mazumder, Ameer Muhammad, Muhammad Imran Nisar, Sitanshi Sharma, Leigh A. Martin, Ravi Prakash Upadhyay, Parul Christian, Maternal BEP Studies Harmonization Initiative, Grace J. Chan, Mulatu M. Derebe, Fred Van Dyk, Luke C. Mullany, Daniel Erchick, Michelle S. Eglovitch, Chunling Lu, Krysten North, Ingrid E. Olson, Nebiyou Fasil, Workagegnehu T. Kidane, Fisseha Shiferie, Tigest Shiferaw, Fitsum Tsegaye, Sitota Tsegaye, Sheila Isanaka, Rose L. Molina, Michele D. Stojanov, Blair J. Wylie, Amare W. Tadesse, Lieven Huybregts, Laeticia C. Toe, Alemayehu Argaw, Giles Hanley-Cook, Rupali Dewan, Pratima Mittal, Harish Chellani, Tsering P. Lama, Benazir Baloch, Mihaela A. Ciulei

**Affiliations:** 1 Department of Nutritional Sciences, The Pennsylvania State University, 110 Chandlee Lab, University Park, PA 16802 USA; 2 Ross and Carol Nese College of Nursing, The Pennsylvania State University, University Park, PA USA; 3 Centre for Health Research and Development Society for Applied Studies, New Delhi, India; 4 Department of Food Technology, Safety and Health, Faculty of Bioscience Engineering, Ghent University, Ghent, Belgium; 5 Department of Pediatric Newborn Medicine, Brigham and Women’s Hospital, Boston, MA USA; 6 Harvard Medical School, Boston, MA USA; 7 VITAL Pakistan Trust, Karachi, Pakistan; 8 Department of Global Health, The George Washington University Milken Institute School of Public Health, Washington, D.C USA; 9 Department of Epidemiology and Biostatistics, Addis Continental Institute of Public Health, Addis Adaba, Ethiopia; 10 Department of Pediatrics and Child Health, Aga Khan University, Karachi, Pakistan; 11 Department of International Health, Johns Hopkins Bloomberg School of Public Health, Baltimore, MD USA; 12 Nepal Nutrition Intervention Project-Sarlahi, Lalitpur, Nepal

**Keywords:** Balanced energy-protein supplementation, Micronutrients, Antenatal, Pregnancy, Lactation, Preconception, Maternal and neonatal outcomes, IPD meta-analysis

## Abstract

**Background:**

Public health and clinical recommendations are established from systematic reviews and retrospective meta-analyses combining effect sizes, traditionally, from aggregate data and more recently, using individual participant data (IPD) of published studies. However, trials often have outcomes and other meta-data that are not defined and collected in a standardized way, making meta-analysis problematic. IPD meta-analysis can only partially fix the limitations of traditional, retrospective, aggregate meta-analysis; prospective meta-analysis further reduces the problems.

**Methods:**

We developed an initiative including seven clinical intervention studies of balanced energy-protein (BEP) supplementation during pregnancy and/or lactation that are being conducted (or recently concluded) in Burkina Faso, Ethiopia, India, Nepal, and Pakistan to test the effect of BEP on infant and maternal outcomes. These studies were commissioned after an expert consultation that designed recommendations for a BEP product for use among pregnant and lactating women in low- and middle-income countries. The initiative goal is to harmonize variables across studies to facilitate IPD meta-analyses on closely aligned data, commonly called prospective meta-analysis. Our objective here is to describe the process of harmonizing variable definitions and prioritizing research questions. A two-day workshop of investigators, content experts, and advisors was held in February 2020 and harmonization activities continued thereafter. Efforts included a range of activities from examining protocols and data collection plans to discussing best practices within field constraints. Prior to harmonization, there were many similar outcomes and variables across studies, such as newborn anthropometry, gestational age, and stillbirth, however, definitions and protocols differed. As well, some measurements were being conducted in several but not all studies, such as food insecurity. Through the harmonization process, we came to consensus on important shared variables, particularly outcomes, added new measurements, and improved protocols across studies.

**Discussion:**

We have fostered extensive communication between investigators from different studies, and importantly, created a large set of harmonized variable definitions within a prospective meta-analysis framework. We expect this initiative will improve reporting within each study in addition to providing opportunities for a series of IPD meta-analyses.

**Supplementary Information:**

The online version contains supplementary material available at 10.1186/s12884-023-05366-2.

## Background

Maternal and child undernutrition continues to be a massive public health issue that needs more attention. Many randomized controlled trials (RCTs) have been conducted in low- and middle-income countries (LMICs) to test the effects of maternal nutrition interventions on pregnancy and postpartum outcomes [1–4]. One such intervention with a growing evidence base is balanced energy-protein (BEP) supplementation. BEP products are ready-to-eat or prepared foods, typically biscuits or powders, that contain energy with a balanced amount of protein intended to supplement the home diet. When designed for use in pregnancy, they often contain micronutrient fortification or a multiple micronutrient supplement in tandem.

Globally, there is a high burden of underweight among women at the start of pregnancy (240 million based on body mass index (BMI) < 18.5 kg/m^2^) [5]. Providing BEP to increase intake of calories and protein during gestation may increase offspring birthweight in undernourished women [4, 6, 7] as well as reduce the risk of stillbirth and small for gestational age (SGA) [4, 7–10]. Further, there could be health benefits for the mother, including improved weight gain [11] and reduced risk of anemia [12, 13]. Beginning in 2016, the World Health Organization (WHO) recommended BEP supplementation during pregnancy in populations with a high prevalence (> 20%) of pregnant women who are undernourished, to decrease the risk of stillbirth and SGA [14]. However, the evidence base informing this recommendation included a wide range of BEP products and nutrient content, creating challenges around implementation.

Over 20 years ago, the need for harmonizing nutrients in an intervention was recognized for micronutrients. An expert panel was convened, and the result was the development of a formulation for a multi-micronutrient supplement for pregnant women from LMICs, called the United Nations International Multiple Micronutrient Preparation (UNIMMAP) [15]. The group recommended that this antenatal supplement, now with a similar nutrition composition, be tested in multiple trials. Similarly, given the heterogeneity in BEP supplements, the Bill and Melinda Gates Foundation (BMGF) convened an expert panel in 2016 to create guidelines for macronutrient and micronutrient composition of BEP supplements [16]. As part of the consultation, the panel explored the “use-case” for BEP distribution and consumption and recommended that next steps include developing the food products and testing the impact on health outcomes among multiple trials.

Following this guidance, the BMGF funded several intervention studies planning to test the effect of BEP in preconception, pregnancy, and/or lactation in countries in South Asia and sub-Saharan Africa. These studies were all designed with BEP supplements that followed the nutrient content targets from the 2016 guidance [17–29], but had independent investigators and were not coordinating together. Studies were specifically funded in multiple countries, given that some heterogeneity across studies (e.g., different environmental factors, diets, and range of nutritional status) is helpful to find answers that can be applicable across diverse populations. However, differences in data and specimen collection techniques, calculations, definitions, and laboratory methods can limit the ability to conduct meta-analyses and compare findings across studies. The need to harmonize protocols has long been recognized but historically occurred through inherently multi-site studies such as the WHO Multicentre Growth Reference study [30] or the Etiology, Risk Factors and Interactions of Enteric Infections and Malnutrition and the Consequences for Child Health and Development (MAL-ED) study [31]. A relatively new framework, called prospective meta-analysis, has emerged to create a process that identifies planned or ongoing studies and forms a collaboration ahead of synthesizing and analyzing pooled data [32, 33].

With forethought on the opportunity to conduct prospective meta-analysis with individual participant data (IPD) from these planned or ongoing studies, the current BEP harmonization initiative was born. The IPD approach allows us to gain insights into BEP effectiveness and the effect of individual-level moderators on treatment outcomes, and the prospective approach allows us to collaborate as a large group and harmonize variables. As described by Seidler et al., “the methodology [for prospective meta-analysis] remains rare, novel, and often misunderstood” [32]. Therefore, our objective in the current paper is to describe the methods of harmonizing data collection and variable definitions and prioritizing research questions for prospective IPD meta-analyses.

## Methods

The Maternal BEP Studies Harmonization Initiative is an ongoing prospective meta-analysis effort that includes harmonizing, optimizing, and enhancing aspects of maternal BEP supplementation studies during pregnancy and lactation for maternal health and infant growth. The methods described here chronicle the initial phase of the initiative to harmonize selected variable definitions (including case definitions and outcomes) and develop research questions for IPD meta-analysis. Methods for individual studies are not part of the current paper but study registry information is included in Table 1, and protocols are or will be published separately by the research team for each study [17–21, 26–29]. The long-term goal of the initiative is to conduct prospective, IPD meta-analyses to examine the effects of maternal BEP supplementation on pregnancy and postpartum outcomes for mother and child.

**Table 1:** Summary information for each randomized controlled trial or effectiveness study in the Maternal BEP Studies Harmonization Initiative

Full study name	Short name	Country	Study design	Life stages receiving BEP	Enrollment @ life stage	Registry	Principal Investigators
Enhancing Nutrition and Antenatal Infection Treatment for Maternal and Child Health	ENAT	Ethiopia	Cluster (health center) and individual (within health center) randomization^a^	Pregnancy	~ 2400 @ < 24 weeks gestation	ISRCTN registry ISRCTN15116516	Anne CC Lee; Yemane Berhane
IMPRINT^b^	IMPRINT	India	Individual randomization	Lactation	816 @ < 1 week after delivery	Clinical Trials Registry-India CTRI/2018/04/013095	Sunita Taneja
Mothers and Infants Nutrition Trial	MINT	Nepal	Individual randomization	Pregnancy	900 @ early pregnancy	ClinicalTrials.gov NCT03668977	James Tielsch
MIcronutriments pour la SAnté de la Mère et de l’Enfant III	MISAME-III	Burkina Faso	Individual randomization with factorial design^c^	Pregnancy Lactation	1788 @ early pregnancy	ClinicalTrials.gov NCT03533712	Patrick Kolsteren
Mumta Pregnant Women Trial	MumtaPW	Pakistan	Individual randomization	Pregnancy	1836 @ pregnancy	ClinicalTrials.gov NCT04012177	Yasir Shafiq; Ameer Muhammad
Mumta Lactating Women Trial	MumtaLW	Pakistan	Individual randomization	Lactation	957 @ < 1 week after delivery	ClinicalTrials.gov NCT03564652	Yasir Shafiq; Ameer Muhammad
Women and Infants Integrated Interventions for Growth Study	WINGS	India	Individual randomization with factorial design^c^	Preconception Pregnancy Lactation	13,500 @ preconception	Clinical Trials Registry-India CTRI/2017/06/008908	Nita Bhandari

*BEP* balanced energy-protein

^a^Pragmatic clinical effectiveness study

^b^Added to Harmonization Initiative in October 2020

^c^Factorial design for MISAME-III was random assignment for pregnancy and random assignment for postpartum; factorial design for WINGS was random assignment for preconception and random assignment for pregnancy (women were also stratified by height at enrollment)

### Participating studies

There are two main approaches for identifying studies for prospective meta-analysis. The first is a systematic search of trial registries, similar to a systematic review of the literature that would be done for a retrospective meta-analysis [32]. The second is to establish inclusive discussions with investigators of planned or ongoing studies, ideally from countries around the world, and set up a collaboration [33]. We followed the second approach because there were already connections between investigators that were funded by the BMGF to examine maternal BEP supplementation, and the study sites represented five different LMIC settings in sub-Saharan Africa and South Asia.

The initiative was funded by the BMGF and began in late 2019. Initially, five principal investigators (PIs) for six studies in Burkina Faso, Ethiopia, India, Nepal, and Pakistan (2 trials) were invited to participate (Table 1). A seventh BEP study in India was added in October 2020, and although the trial had recently completed and would not be able to change data collection, it was closely aligned with the other studies and some harmonization was still possible through IPD meta-analysis. Also, the PI of the added trial was already part of the harmonization initiative as a co-investigator on another trial.

### Ethics

Each study carried out methods in accordance with relevant guidelines and regulations and all protocols were first approved by the appropriate ethical boards, locally and at the home institution of the PI (when different from the site location). Informed consent was obtained from all study participants and details are published by each individual study. The harmonization work of the initiative, described here, was solely with study protocols and did not include any human data. Our future meta-analysis will be conducted with de-identified data and has been deemed “not human research” by the Pennsylvania State University Institutional Review Board (STUDY00017249).

### Initiative members

This initiative included the investigators from individual trials, a technical advisory group (TAG), and a coordinating team. Among the main contributors, the study PIs are considered the decision makers, and collectively make the final call on aspects of harmonization. The TAG was established with five experts in maternal and child nutrition research in LMICs, and they are consulted on an ongoing basis for input and advice. The coordination and management team was created under the direction of Dr. Gernand at Penn State, an investigator with experience in maternal nutrition trials in LMICs but external to the current BEP studies. The coordination team plans and guides the work, ensuring the initiative moves forward, and synthesizes and investigates details. The BMGF has served in a facilitation role throughout, including funding and organizing the workshop described below.

Participating members of this initiative covered a wide range of research, laboratory, and clinical expertise. Individuals included maternal, obstetric, perinatal, and pediatric clinician-researchers, pregnancy; lactation, and nutrition experts; and trialists, statisticians, and epidemiologists with representation across North America, Europe, sub-Saharan Africa, South Asia, and Australia.

### Workshop

After key members were identified and recruited, activities began and continue to the present. The main activities included an in-person workshop, remote meetings, and surveys – all conducted with members of the initiative (co-authors on this manuscript). The two-day workshop was held at the BMGF headquarters in Seattle, Washington in February 2020. In addition to initiative members described above, researchers from around the world were in attendance, including experts in the fields of nutrition, epidemiology, the gut microbiome, biomarker discovery, neurodevelopment, pregnancy, fetal development, and pediatrics.

The workshop had three main goals, each addressed in a separate, facilitated session:

1. Create a harmonized variable list with common definitions
2. Align on a harmonized protocol for biospecimen collection and analysis
3. Discuss the potential for harmonized neurodevelopment assessment

Ahead of the workshop, the coordination team collected all study protocols and created charts and tables to document the pre-existing similarities and differences. These files were shared at the workshop with all participants. During the workshop, large posters were displayed to aid in mapping out harmonized variable definitions. Posters included existing definitions and measurement protocols from each study and existing or standard definitions from the literature and authoritative health agencies or professional societies along with space to draw out new ideas.

Substantial progress on all three goals was achieved and next steps were discussed. Throughout the workshop, teams expressed an interest in creating a network with regular meetings to discuss problems encountered and operational difficulties in addition to the harmonization work. At the end of the workshop, harmonization on the initial variables list was in place and a list of additional variables to align was created. Harmonizing biospecimen collection and neurodevelopment assessment also occurred but is not part of the current paper because the onset of the COVID-19 pandemic caused changes and reductions in the ability of most trials to collect biospecimens and/or continue with plans for neurodevelopment assessment. Further, other groups and laboratories were funded to measure and examine these components.

### Follow up meetings

From March to May 2020, regular, remote meetings occurred (via Microsoft Teams) approximately every 2 weeks to discuss additional variables and details of harmonization. From the end of May to mid-July there was a pause on group meetings to update the reports and variables. From August 2020 to the present, meetings occur every 4 to 8 weeks. TAG members were consulted outside of regular group meetings due to the difficulty of finding a common meeting time for the large number of people involved, and because it was useful to get input from TAG members separately.

Surveys were utilized between meetings to query teams individually and allow feedback ahead of (and uninfluenced by) group discussions. Survey topics included degree of agreement with proposed variable definitions, the need for more discussion of variables either individually or as a group, and the feasibility and use of newly created variable definitions. These surveys were key to coordination, planning efficient remote meetings, and collecting detailed information from teams.

### Similarities and differences between studies

In the preliminary assessment of overlap between studies, similarities and differences were identified. Per the goal of the initiative, all studies were testing BEP supplementation compared to a control group that did not receive BEP (but did receive the standard of care for pregnancy/lactation/infancy). All studies were collecting maternal and infant anthropometry, and all studies were using ultrasound-based gestational dating during pregnancy. Primary outcomes were similar across studies: infant size at birth for pregnancy studies and infant size and growth velocity (change in weight or length) at 6 months for lactation studies. We identified the following major differences across studies: the randomization design (individual vs. cluster, different number of intervention arms), other interventions coupled with BEP, timing of enrollment during pregnancy (early vs. mid), timing of anthropometry measurements, and timing and number of biospecimens collected.

### Harmonization of variables

At the workshop, a total of nine outcome variables (or variable clusters) were originally slated for discussion in three breakout groups with representation from each study team and at least one member of the TAG: birth weight, birth length, birth head circumference, infant anthropometry, maternal anthropometry, stillbirth, infant mortality, maternal mortality, and gestational age. This first set of variables was selected based on overlap of outcomes that were primary or secondary objectives of individual studies or important rare outcomes for which IPD meta-analysis would allow estimation of the effect of BEP.

Each workshop session brought about new ideas for additional variables to collect or harmonize across sites. Examples that were brought up in multiple sessions included: SGA, short-for-gestational age, large-for-gestational age, preterm birth (spontaneous versus induced), preeclampsia, and causes of maternal death. There were also multiple discussions around capturing the details of delivery (e.g., Cesarean section), maternal morbidities, and corresponding international classification of diseases (ICD) codes (although we did not link ICD codes to variables). These workshop discussions formed agendas for variables to discuss in the first set of remote meetings, and during the months of remote meetings, we continued to work through discussions of variables that the group determined to be important and feasible to harmonize. Priority was given to maternal and infant health outcomes; key variables that help to understand or describe the outcomes (e.g., gestational age, mode of delivery) or intervention (e.g., nutrient intake, food insecurity); and variables identified as potential effect modifiers (e.g., pre-pregnancy BMI).

For each variable, we considered a range of details to harmonize including timing, measurement tool, number of measurements, and quality control. Loosely, our framework for alignment at this stage covered levels 1–3, and occasionally level 4, for details to include for outcomes in a ClinicalTrials.gov registry [34]. For example, newborn anthropometry was like a domain, one of the specific measurements was birth weight (with details on how to conduct the measurement), and the specific metric was the gestational age- and sex-specific z score, if drawing parallels with the ClinicalTrials.gov outcome levels.

Ultimately, more than sixty variables were reviewed, discussed, and a group consensus reached for a definition and variable name (Supplementary Table 1). These harmonized variables generally fall into eight domains/categories: anthropometry, pregnancy characteristics, pregnancy complications, labor and birth outcomes, mortality, food insecurity and infant feeding, maternal dietary intake, and supplement adherence (compliance). After our variable harmonization process, we were made aware of the core outcome measures in effectiveness trials (COMET) initiative, which establishes core (or minimum) sets of outcomes for specific areas of health [35]. To our knowledge, the main related COMET set is the pregnancy and childbirth standard set [36]. Our developed harmonized variables are minimally overlapping with these because our variables are specific to LMIC settings and focused on those needed for studies of BEP supplementation while theirs were established for studies evaluating perinatal care.

### Variables beyond the scope of harmonization

During meetings, there were many variables discussed in relation to the planned IPD meta-analyses that the group decided not to harmonize. In some cases, variables were too complicated or different across study settings (e.g., socio-economic status). In other cases, it seemed like variables were too far outside the goals of the IPD meta-analysis, and spending time to align definitions would likely not improve the IPD meta-analyses in a meaningful way (e.g., sepsis). There was a desire across study teams to harmonize adherence to the intervention, but decisions on the best way to measure adherence were not clear and certain field methods in use, e.g., direct observation of supplement consumption, were not feasible in all settings. Variables that were considered but ultimately not harmonized included: wealth index, socio-economic status, maternal education/literacy, maternal or infant sepsis, maternal postpartum hemorrhage, severe features of preeclampsia, and additional details of labor and delivery (beyond those in Supplementary Table 1). Finally, maternal postpartum depression was planned for each study, using either the Edinburgh Postnatal Depression Scale [37] or the Patient Health Questionnaire-9 [38] depression interview. While there was group discussion about these depression scales, including field practices and advice on their use, we did not decide to harmonize a depression variable particularly because data collection was already in progress using the different scales.

As in traditional meta-analysis, we still plan to use variables that are defined in different ways (i.e., not harmonized), and we will leverage the full datasets to create variables that are as closely aligned as possible. For example, with socio-economic status, we can use composite indices created within each trial to combine tertiles or quartiles of income/wealth.

### Research questions for IPD meta-analyses

The focus of the harmonization effort has been planning for IPD meta-analyses. Early in the process, the full group discussed, vetted, and prioritized objectives for a series of IPD meta-analyses. We wrote these out in the form of research questions, such as, *what is the effect of BEP supplementation during pregnancy on the risk of small-for-gestational age?* Or *what is the effect of BEP supplementation during lactation on infant length at 6 months of age?*

The objectives clustered into four groups:

1. Common or continuous outcomes (those that studies are individually powered to address)
2. Rare outcomes (those that studies are not individually powered to address)
3. Harmonized sub-studies (not discussed in the current paper)
4. Stratified analyses (to identify groups where BEP could provide the largest benefit)

Common and rare outcomes are listed in Table 2 by the life stage of BEP supplementation. For stratified analysis, the following effect modifiers were selected to examine: maternal education, age, parity, height, BMI, and mid-upper arm circumference, adherence to BEP, geography, and household-level food insecurity.

**Table 2:** Maternal and infant outcomes prioritized for IPD meta-analysis during the harmonization process

	Pregnancy and birth outcomes (affected by BEP during pregnancy)	Postpartum outcomes (affected by BEP during pregnancy and/or lactation)
Common or continuous outcomes	Infant size at birth (weight, length, small for gestational age, short for gestational age)	Infant anthropometry at 6 months of age
	Maternal gestational weight gain	Infant growth velocity from birth to 6 months of age
	Maternal postpartum BMI and MUAC	Maternal postpartum BMI and MUAC
	Maternal anemia and IDA in the third trimester	Maternal postpartum anemia and IDA
	Maternal inflammation in the third trimester	Maternal postpartum inflammation
Rare outcomes	Maternal mortality	Maternal mortality
	Stillbirth	Perinatal, neonatal, and infant mortality
	Preterm birth	
	Gestational hypertension/Preeclampsia	

*Abbreviations*: *BMI* body mass index, *IDA* iron deficiency anemia, *MUAC* mid-upper arm circumference

## Discussion

### Lessons learned

We describe here the initial process for a prospective meta-analysis of seven LMIC studies across five countries testing BEP intervention during pregnancy and/or lactation to improve maternal and infant outcomes. This collaborative work leveraged and integrated knowledge generation beyond that which would have been achievable by individual studies alone. Such efforts are challenging to undertake outside of “multi-country/site” studies and across multiple PIs, timelines, and independent grant mechanisms, but are becoming more common [39–42]. This harmonization effort found extremely collaborative investigators and a coordination team supported by a visionary funder. Important elements had to come together in the early stages of grant-making to make this work possible. An in-person workshop combined with ongoing meetings and surveys allowed for open communication and prioritization of the work. The result is a core set of over sixty harmonized variables.

### Prior harmonization and standardization efforts

Researchers and clinicians have long tried to raise awareness about the need for standard definitions and protocols, particularly for maternal and infant health [43, 44]. The COMET initiative was established in 2010 to develop “core outcome sets” to extend the concept of standardization to establish a minimum set of outcomes to measure for health conditions [45]. Standard definitions are commonly published and reviewed by authoritative bodies such as the WHO, the Centers for Disease Control and Prevention, the American College of Obstetricians and Gynecologists, and the Brighton Collaboration, and we consulted these sources as a starting point for all variables. We also looked at other global collaborations, such as the International Network of Obstetric Survey Systems (INOSS) for the study of rare pregnancy problems [46], the Global Pregnancy Collaboration (CoLab) for the study of preeclampsia [47], and the recently created International Milk Composition (IMiC) consortium (https://www.milcresearch.com/imic.html) for the harmonization of methods for human milk analysis (two of the studies in this group are also participating in IMiC).

### Ongoing work during the COVID-19 pandemic

The harmonization process has been highly interactive, with open sharing between investigators. The group has benefited from the incredible depth of knowledge from the research teams’ members and the group of advising experts. Many researchers involved in this initiative have decades of experience in running nutrition trials in LMIC settings. The COVID-19 pandemic presented enormous challenges for the individual studies and field work, but minimal disruption for the initiative. Participation levels were quite high at the beginning of the pandemic and have remained as such. Effective remote meetings were possible via Microsoft Teams, particularly by sharing slides that detailed discussion topics and questions and by using the chat feature in addition to vocal comments. We share a list of lessons learned in Table 3.

**Table 3:** Key lessons learned from the process of harmonizing variables across different studies of BEP intervention in LMICs

Broad Lessons	Specific examples^a^
**Study level**	
Harmonization of variables should include both: a) aligning existing variables and b) deciding which measurements should be captured in all studies	While we started by harmonizing variables that were already part of each study (e.g. newborn weight), we also discussed variables that would be important to capture in every study that were not currently present (e.g., food insecurity, certain labor and delivery outcomes).
Quality control should be part of harmonization, but it is particularly difficult to align (especially after studies have started)	We aimed to align quality control procedures for anthropometry and ultrasound variables but ultimately had to refer to best practices from reliable sources as a goal and allow each study to follow quality control practices within their specific field constraints.
Some methodological issues cannot be completely harmonized, but differences can be documented to aid interpretation	For newborn length, we agreed that having two blinded measurers was the ideal practice, but some studies had the capacity for only one measurer.
Some variables do not fit well with harmonization, and decisions on handling these variables in IPD meta-analysis can be made at the analysis level	The structure of education varies considerably across countries, including names for different levels/classes. All studies were collecting information about the amount education each participant completed, and we will reconcile these data during analysis
Ethical and cultural values affect what is allowed and approved in different settings	Harmonization of estimated fetal weight in the third trimester was desired to examine in utero effects of BEP on fetal growth, however in one country, this measurement was not allowed by the IRB due to the inability to address growth restriction in clinical care if identified.
**Variable level**	
There are many layers to harmonizing variables that should be discussed – equipment, training of staff using equipment, calibration of equipment, quality control, number of measurements, timing (e.g., in gestation) of measurement, and handling of data (e.g., cut-offs)	Anthropometry and gestational age were the two variable groups with the most layers to discuss. We decided to harmonize some layers (which measurements were taken, e.g. birth weight, length, and head circumference) but could not align others (expertise of staff taking ultrasound measurements).
Some variables are quite complicated, and take much more time to work out	Gestational age is measured in different ways depending on the timepoint in pregnancy and the available resources. The group spent considerable time reviewing different components of ultrasound measurements and equations to translate measurements to gestational age and ultimately decided to use INTERGROWTH-21st equations (for early and late gestation) but developed an altered protocol for deciding which measurements to capture.
Creating new names for variables (with the same underlying intent) can aid group consensus	We had a lot of discussion about how to define stillbirth and miscarriage. As the WHO defines stillbirth as ≥28 weeks, the group still wanted to capture loss below 28 weeks but did not want to call it miscarriage. So, we decided to create categories by gestational age < 28 weeks and call each “fetal loss <X weeks”.
Creating proxy variables can aid harmonization, particularly when is not possible to capture a clinical variable with standard diagnostic tests (or not possible to capture it at all)	We wanted to capture cephalopelvic disproportion as a safety assessment, however components of this outcome in clinical obstetric definitions were not possible to obtain in these settings. We created a proxy variable, named “maternal-fetal disproportion”, to capture available data with the same underlying meaning (that the baby was too large to fit through birth canal).
When “perfect” capture of clinical variables is not possible, divide into two variables: a) ideal measurement by study team and b) clinical diagnosis from medical record (even if you cannot obtain details on how the diagnosis was made)	Preeclampsia currently has a clinical definition that includes many severe signs and symptoms that are not possible to capture in these studies. We decided to create a definition feasible for study teams to measure (high blood pressure and proteinuria) and a separate variable to capture clinical diagnosis from the medical record (which will vary by location).

^a^Supplementary Table 1 has additional details about each variable or set of variables mentioned here

While key variables have been harmonized, some final questions (e.g., denominators for mortality rates) will continue to be discussed for analysis. Next steps in the work include establishing a composite data dictionary, developing detailed data analysis plans, and conducting the series of IPD meta-analyses. These steps will require further discussion of variables, particularly those that may be included in analysis but that were not harmonized (e.g., maternal education).

### Pros and cons of the harmonization phase

This harmonization initiative has many strengths. As previously discussed, the wide-ranging and long-standing expertise of investigators within each individual study was critical during the vetting and final decision making of variable definitions. Often there was an important consideration that was prompted by a single group member. The TAG, as a separate body from the study investigators, strengthened the process by providing insight from experts that were not currently conducting BEP studies. Additionally, a coordination and management team was central in keeping the harmonization process going, keeping the large group connected and engaged, and doing necessary background work to glean details important for complicated or difficult to define variables.

We encountered several challenges, some of which we could not resolve fully. While we maintained high standards for developing rigorous, detailed definitions of variables, there were three key scenarios which may affect our final analysis: 1) investigators agreed on a definition knowing that it was ideal but that not all studies could meet the definition, 2) investigators agreed on a definition that was not ideal, but that was practical across settings, and 3) investigators agreed that a common definition was too hard to reach across studies (often due to the level of clinical information available). Sometimes it was very difficult to reach agreement, and in these cases, the coordination team gathered individual input (which was kept anonymous) and re-visited details of other published definitions and protocols to present new information and choices to the whole group. In every case, a solution was agreed upon. If similar opportunities arise for other analogous studies, we highly advocate for a harmonization effort to be conducted and have key recommendations (Table 4).

**Table 4:** Recommendations for future harmonization efforts

• Begin harmonization before studies start enrollment	
• Start by establishing specific aims (with key outcomes) for the IPD meta-analysis – this should drive the priority for the variables to harmonize	
• Create a roadmap of the whole process from start to finish, including all categories of variables to harmonize	
• Schedule more discussion and deliberation for more complex variables	
• Include a coordinating team and an outside technical advisory group	
• Use multiple modes of communication and interaction	

## Conclusion

In our harmonization effort, as part of an initiative for prospective IPD meta-analysis of pregnancy and lactation studies, we had highly collaborative investigators, along with a coordination team and TAG that facilitated detailed technical conversations and agreement on variable definitions across diverse country settings. Among numerous investigators for seven RCTs of maternal BEP supplementation in five different LMICs, we successfully harmonized over sixty variables related to maternal and infant health. We hope this work is a valuable resource to other researchers and clinicians as we collectively work to improve the health of mothers and infants around the globe.

## Supplementary Information

**Additional file 1: Supplementary Table 1.** Harmonized variables with definitions, rationale, and additional considerations.

## Data Availability

Not applicable.

## Abbreviations

BEP: Balanced energy-protein supplementation
BMGF: Bill and Melinda Gates Foundation
BMI: Body mass index
CoLab: Global Pregnancy Collaboration
ICD: International classification of diseases
INOSS: International Network of Obstetric Survey Systems
IMiC: International Milk Composition
IPD: Individual participant data
LMIC: Low- and middle- income countries
MAL-ED: Etiology, Risk Factors and Interactions of Enteric Infections and Malnutrition and the Consequences for Child Health and Development
PIs: Principal investigators
RCT: Randomized controlled trial
SGA: Small for gestational age
TAG: Technical advisory group
UNIMMAP: United Nations International Multiple Micronutrient Preparation
WHO: World Health Organization
