# Supplementary material for: Harmonization of maternal balanced energy-protein supplementation studies for individual participant data (IPD) meta-analyses – finding and creating similarities in variables and data collection
Source: BMC Pregnancy Childbirth. 2023 Feb 11;23:107. doi: 10.1186/s12884-023-05366-2 (PMC9919738; doi:10.1186/s12884-023-05366-2)
Supplement: Supplementary file 1 — Additional file 1: Supplementary Table 1. Harmonized variables with definitions, rationale, and additional considerations. [file 12884_2023_5366_MOESM1_ESM.docx]

**Supplementary table 1.** Harmonized variables with definitions, rationale, and additional considerations.

| Variable | Definition | Rationale | Additional Considerations |
| --- | --- | --- | --- |
| ANTHROPOMETRY | | | |
| Birth Weight | One measurement in grams to be collected as soon as possible within 72 hours of birth by a single measurer using any digital scale precise within 10g and calibrated daily (1). If two measurements are used, they must be within 50 grams or a second set of weight measurement is taken (then a third, if needed). All weight measurements should be recorded in the database. | Adapted from INTERGROWTH-21^st^ procedures (1). | Each study will attempt to closely follow quality control and training/re-training protocols from INTERGROWTH-21^st^ for all anthropometric measurements at birth. We will not establish quality control procedures specifically for this initiative.    A longitudinal newborn birthweight sub-study is outlined below. |
| Low birth weight (LBW) | Birth weight <2500 g    Incidence: % of live births that weigh <2500 g | WHO definition (2)    Using only live births because stillbirths are not weighed in many settings |  |
| Birth Length | Two (or more) measurements in centimeters to be collected as soon as possible within 72 hours of birth by two blinded measurers or a single measurer, with a trained assistant (1). The measurements should be within 7 mm or a second set of measurements is taken and checked (then a third, if needed). All length measurements will be recorded in the database. | Adapted from INTERGROWTH-21^st^ procedures (1)    Not all studies have the capacity to use two independent measurers, so we agreed that at least two measurements would be taken (even if by the same person). | Record the measurement to the last *completed* mm (not the nearest mm) |
| Stunting | <-2 SD length for age z score | WHO Child Growth Standards (3) |  |
| Birth Head Circumference | Two (or more) measurements in centimeters to be collected as soon as possible within 72 hours of birth by two blinded measurers or a single measurer with an assistant (1). The measurements should be within 5 mm or a second set of measurements is taken and checked (then a third, if needed). All head circumference measurements should be recorded in the database. | Adapted from INTERGROWTH-21^st^ procedures (1)  Not all studies have the capacity to use two independent measurers, so we agreed that at least two measurements would be taken (even if by the same person). | Record the measurement to the last *completed* mm (not the nearest mm)    Training on measurement with molding was determined to be high priority for standardization across sites, and a specific protocol from one of the studies was shared with the group. |
| Infant Anthropometry | Length, weight, head circumference, and MUAC will be measured for infants at 1, 3, and 6 months of age, following the same protocols at birth for length, weight, and head circumference, and following the WHO protocol for MUAC (2 measurements, recorded to last *completed* mm). | Other timepoints were discussed, but it was concluded that following infants past 6 months should be optional for the purposes of the pooled analysis. Some studies are collecting chest circumference, but it was not discussed as a measurement to harmonize. | Each study will attempt to closely follow quality control and training/re-training protocols from INTERGROWTH-21^st^ and WHO for all anthropometric measurements across infancy. We will not establish quality control procedures specifically for this initiative. |
| Maternal Anthropometry | Height, weight, and MUAC will be recorded at enrollment; timing of weight and MUAC measurements across pregnancy is determined within each study. Repeat height measurements are optional but encouraged when studies have a high proportion of teenage pregnancies.    Two (or more) measurements should be taken for each parameter, and all measurements should be recorded in the database. Weight should be measured on a digital scale with no shoes and light clothing. It was agreed that in cold season that outer layers should be removed. | Earliest and latest possible pregnancy measurements will allow for calculation of gestational weight gain.    Postpartum weight is planned in each study but is not part of pooled analysis plan. | Measure weight at screening or during pre-pregnancy tracking if possible in order to have a weight for pre-pregnancy BMI calculation (this will not be possible in most studies)    There was discussion about stratification by maternal height for randomization. Individual studies may choose to do this, but the group decided to not harmonize on this.    Each study will attempt to closely follow quality control and training/re-training protocols from WHO for all maternal anthropometric measurements. We will not establish quality control procedures specifically for this initiative. |
| Gestational Age | Determination of gestational age will be ultrasound-based using the INTERGROWTH-21^st^ equations and protocols for measurement (4,5).    All studies will collect the date of ultrasound, the raw measurement values, and store the digital images.    CRL will be the first measurement attempted in all scans. If CRL is 15-95 mm, record CRL. If CRL >95 (or is impossible to measure because the fetus is too large to fit within the field of view) HC and FL will be measured and recorded. If CRL is 85-95 mm, measure FL and HC (in addition to CRL). Whenever FL and HC are taken, also measure and record BPD (if possible).    INTERGROWTH-21^st^ equations will be used with CRL (early pregnancy) (6) or FL and HC (mid- to late pregnancy) (7) to calculate gestational age.    Each study will conduct real-time QC of 10-15% of images (internal to study). | CRL was agreed upon as the first measurement to standardize the procedure, knowing that LMP has a range of error and if the technician performing the scan thinks the gestational age is later, she/he might take BPD, etc. without ever trying CRL (and in some cases, CRL would have been within range). The overlap in taking CRL, FL, HC, and BPD measurements was agreed upon for several reasons: 1) previous recommendations suggested using BPD, etc. when CRL was >85 mm, 2) CRL, FL, HC, and BPD are all valid measurements in this gestational age window, and 3) as this is a challenging time to obtain fetal CRL, other measurements will provide “back-up” if quality control checks deem any individual measurement is not correct and cannot be used. | The number of repeat measurements (i.e., 2 or 3) to capture was not harmonized, as there were too many other factors to align for ultrasound.    Each study will attempt to closely follow measurement procedures, quality control, and training/re-training protocols from INTERGROWTH-21^st^ ultrasound measurements.    Measurement of BPD as outer-inner (rather than outer-outer) was discussed because some studies are using this technique. We reviewed unpublished data which found that differences in gestational age estimation across the two measurements is negligible. Also, BPD is not part of the primary equation for gestational age estimation in late pregnancy. Therefore, we did not harmonize the measurement of BPD.    Enrollment based on ultrasound-estimated gestational age was discussed but determined to be unnecessary. A late gestation ultrasound will be collected by some studies to estimate fetal weight.    Transcerebellar diameter was discussed but because it is only applicable after 24 weeks and studies are doing ultrasound for gestational dating earlier than this, we decided transcerebellar diameter was not necessary to collect across studies.    External quality control (expert reviewer to examine images across studies) was discussed and the group decided not to proceed because 1) logistical challenges and 2) several studies were too far along. |
| Estimated fetal weight | Based on AC in cm and HC in cm  log(EFW) = 5.084820 − 54.06633 × (AC/100)^3^ − 95.80076 × (AC/100)^3^ × log(AC/100) + 3.136370 × (HC/100)  from 32-36 weeks gestation | INTERGROWTH-21^st^ equation (8) with a shorter gestational age window to more closely standardize collection across studies. | A wider gestational age window was considered but it was agreed that the 32-36 week gestational age range was best for comparison across studies. |
| Small for gestational age (SGA) | Weight <3^rd^ and <10^th^ percentile for gestational age (and sex) using INTERGROWTH-21^st^ Growth Standards | INTERGROWTH-21 Growth Standards (9) and WHO definition (10) | Captured over two variables: <3^rd^ percentile and <10^th^ percentile |
| Short for gestational age (ShGA) | Length <3^rd^ and <10^th^ percentile for gestational age (and sex) using INTERGROWTH-21^st^ Growth Standards | INTERGROWTH-21 Growth Standards (9) | Captured over two variables: <3^rd^ percentile and <10^th^ percentile |
| Large for gestational age (LGA) | Weight >97^th^ and >90^th^ percentile for gestational age (and sex) using INTERGROWTH-21^st^ Growth Standards | Adapted from Xu, 2010 (11) to also include a 90^th^ percentile cut-off | Captured over two variables: >97^th^ percentile and >90^th^ percentile |
| PREGNANCY CHARACTERISTICS | | | |
| Parity | Self-reported number of births (no gestational age cut-off, but the variable is intending to include pregnancies that reach 22 weeks or greater) | Following DHS survey methods (12)    Includes all births (live birth and stillbirth) but is not intended to include miscarriage | It was agreed that most women would not readily recall if a previous pregnancy had reached a certain gestational age (e.g., ≥22 weeks). |
| Pre-pregnancy weight | Measured within 1 year before conception (ideal) or measured <14 weeks gestation | INTERGROWTH-21^st^ used maternal weights <14 weeks gestation if pre-pregnancy weight was not available (8) | Self-report will not be included due to reliability concerns. |
| Gestational weight gain | Rate = kg gained per week (usually by trimester or within discrete intervals)    If “pre-pregnancy” weight available:  Current weight – pre-pregnancy weight    Total = final weight before delivery – pre/early-pregnancy weight | Following IOM Pregnancy Weight Guidelines (13)    Rate of weight gain in second half of pregnancy can be examined across studies, even if some studies are not able to capture pre-pregnancy weight | May also examine weight gain as a percentage (weight gain/pre-pregnancy weight) |
| PREGNANCY COMPLICATIONS | | | |
| Gestational hypertension | Blood pressure elevation of ≥140 mm Hg systolic OR ≥90 mm Hg diastolic on 2 occasions at least 1 hour apart    After 20 weeks of gestation (no preexisting hypertension)    In the absence of proteinuria | Adapted from ACOG (14) for field conditions. Timing between measurements is difficult in these settings. It was agreed that 1 hour was sufficient, but a 2^nd^ measurement taken at a subsequent visit (e.g., the following week) would also be acceptable. | BEP formulation includes supplemental calcium, increasing the importance of this variable. |
| Preeclampsia (diagnosed by measurements from study staff) | Gestational hypertension (after 20 weeks gestation)  AND  1) Proteinuria (2+ on urine protein dipstick)    Note: may miss preeclampsia during labor when study staff are not present | Adapted from ACOG (14) for field conditions | BEP formulation includes supplemental calcium, increasing the importance of this variable. The group decided not to attempt to include classification of preeclampsia with severe features due to problematic nature of collecting this data. |
| Preeclampsia (diagnosed clinically) | Diagnosed by clinicians providing antenatal or intrapartum care  Includes any diagnosis whether or not designations of mild/severe or specifics of diagnosis criteria are known | Some studies will have medical record data but will not measure blood pressure and proteinuria directly. | We discussed not including this variable, but it would mean that some studies would be missing preeclampsia. The group agreed that clinically diagnosed preeclampsia would be useful to capture, but to separate it from preeclampsia classified by study measurements. |
| Maternal anemia | During pregnancy  **1^st^ & 3^rd^ trimester** (all): Hb <110 g/L  Moderate: Hb 70-99 g/L  Severe: Hb <70 g/L  **2^nd^ trimester*** (all): Hb <105 g/L  Moderate: Hb 65-94 g/L  Severe: Hb <65 g/L  (*based on ~5 g/L decline in Hb in the 2^nd^ trimester)    Postpartum/Non-pregnant  All: Hb <120 g/L  Moderate: Hb 80-109 g/L  Severe: Hb <80 g/L | WHO definition (15) | Evaluation of cut-points for anemia in pregnancy is ongoing and we will adjust these definitions as appropriate. |
| Maternal iron deficiency | Define by  **Ferritin** <15 ng/mL  and (separately)  **Soluble transferrin receptor** (sTfR) (cutpoint undecided)    Both biomarkers need to be adjusted for inflammation (using CRP and AGP) (16) | Both biomarkers are affected by inflammation. Measuring both ferritin and sTfR will allow calculation of the body iron index (total body iron) and the sTfR index. | May not be possible in some studies collecting VAMS.    Will require measurement of CRP and AGP (the group was trying to avoid more measurements but these will be needed). |
| Maternal iron deficiency anemia | Anemic and iron deficient (per definitions above) | The group was particularly interested in quantifying the proportion of women with anemia due to iron deficiency |  |
| LABOR AND BIRTH OUTCOMES | | | |
| Live birth | Infant with any signs of life after birth (e.g., crying, breathing, movement) | Need to distinguish live from non-live birth across gestational ages | In the hospital setting, evidence of life may include heartbeat, pulsation of umbilical cord, or Apgar score >0. Most studies will not have the ability to assess Apgar score. |
| Preterm birth (PTB) | Live birth <37 completed weeks    (and very preterm birth: live birth <32 completed weeks) | WHO definition (17) |  |
| Mode of delivery | 1. Vaginal 2. Assisted vaginal (forceps or vacuum) 3. C-section |  |  |
| C-section indication | 1. Obstructed/prolonged labor 2. Fetal presentation (e.g. breech) 3. Cord prolapse 4. Fetal intolerance of labor 5. Maternal hypertension/pre-eclampsia, eclampsia 6. Prior c-section (repeat) 7. Elective (planned) 8. Other _______ | The group wanted to collect indication mainly to estimate occurrence of obstructed labor | Not all groups will be able to collect data for each of the listed indications, and some will have additional indications. There will be consideration in data analysis for collapsing some categories if needed, but more detail is preferred when possible. |
| Onset of labor | 1. Spontaneous – contractions started on their own 2. Induced – intervention used to start contractions 3. None – c-section without start of labor |  |  |
| Rupture of membranes (ROM) | 1. Spontaneous – membranes ruptured on their own 2. Artificial – intervention used to rupture membranes |  |  |
| PROM (premature rupture of membranes) | Spontaneous rupture of membranes before onset of labor |  |  |
| PPROM (preterm premature rupture of membranes) | Spontaneous rupture of membranes before onset of labor at < 37 completed weeks |  |  |
| Spontaneous preterm birth | Birth before 37 completed weeks (37 0/7 weeks) after spontaneous labor |  |  |
| Labor augmentation | Anything done to help labor progress (e.g. oxytocin, artificial ROM) |  | Preferably, details on what was done/used will be recorded |
| Obstructed labor (proxy) | Any maneuvers or instruments (e.g. forceps) were needed to help deliver the baby  OR c-section due to obstructed/prolonged labor |  | Preferably, details on what was done/used will be recorded |
| Maternal-fetal disproportion | Obstructed labor (proxy)  AND  [LGA  OR short mother <145 cm in stature  OR head circumference >2 z score at birth (accounting for molding)  OR clinically diagnosed CPD  (cephalopelvic disproportion)] | The purpose of collecting this variable, as a proxy for CPD, was discussed for safety assessment.    LGA and short mothers are two factors that could contribute to CPD, even though not part of a traditional definition |  |
| Preterm delivery | Any birth outcome ≥22 and <37 completed weeks | To include/count stillbirths as part of the burden of preterm |  |
| MORTALITY | | | |
| Fetal loss <22 weeks | Fetal death <22 weeks gestation (spontaneous) | Not consistent with WHO use of the term miscarriage, but follows the time period before WHO classified pregnancy loss as perinatal mortality (18) | Often called miscarriage or spontaneous abortion |
| Fetal loss <28 weeks | All fetal deaths <28 weeks gestation | While not originally planned for discussion, the group felt that it was important for all studies to capture any pregnancy loss, not just those ≥28 weeks gestation. | Accounts for what some call miscarriage and would be the other category for all loss before stillbirth (as defined by WHO). |
| Fetal loss 22 to <28 weeks | Fetal death ≥22 weeks and <28 weeks gestation | WHO definition (2) | Often called early stillbirth or late miscarriage. |
| Stillbirth | A fetal death when the gestational age is ≥28 weeks.    A fetal death will be defined as being born without evidence of life (see live birth definition above). | Follows the WHO definition for gestational age but drops the infant weight criterion (19). While there was discussion around capturing the weight of stillborn babies, it was determined to be impossible in these settings. | Some studies capture the sex of stillborn babies, but it was not determined to be essential to the harmonization work. |
| Perinatal mortality | Number of stillbirths (≥28 weeks gestation) and deaths in the first week of life (early neonatal mortality) | Adapted from WHO definition (18) to meet our harmonized stillbirth definition (increased from ≥22 to ≥28 weeks). |  |
| Infant Mortality | Any infant death under one year of age (2). An agreement was reached to also capture cause of death (18), date of death, and time of death (if death is within 7 days of birth). | Following WHO definition (2) and WHO verbal autopsy (18) | Comparison of verbal autopsy procedures were not specifically compared beyond agreement that the WHO process was followed. |
| Neonatal mortality | Deaths among live births during the first 28 completed days of life (0-27 days). | WHO definition (2) |  |
| Maternal Mortality | Maternal death from any cause related to or aggravated by pregnancy or its management (excluding accidental or incidental causes), during pregnancy and childbirth, or within 42 days of termination of pregnancy (18). | Following WHO verbal autopsy (18) |  |
| FOOD INSECURITY AND INFANT FEEDING | | | |
| Household Food Insecurity | Categorize food secure and mild/moderate/severe food insecurity using the Household Food Insecurity Access Scale (HFIAS) at enrollment | HFIAS is a tool designed for global use. It is already being used by several studies.    Main purpose is to estimate food insecurity across contexts | Scores and other indicators that can be created from the HFIAS tool could be added to the analysis. |
| Prelacteal feeding (<72 hours) | Infant receives any liquids of foods other than breast milk, vitamins, minerals, or medicines within the first 72 hours of life (e.g., honey, ghee, boiled water, goat’s milk, ritual fluids). | Use of prelacteal feeding is common in many settings and should be captured. | Infants will still be considered exclusively breastfed at later timepoints even if prelacteals are given. |
| Exclusive breastfeeding | Infant receives breast milk and nothing else except vitamins, minerals, or medicines (e.g., ORS, drops, or syrups). Breast milk can be breast or bottle fed and from mother or wet nurse. | Adapted from WHO definition | WHO definition is based on recall of the previous day, but not all BEP studies are collecting data in this way (some are collecting monthly information, which prevents the overestimation that occurs with one-day recall)    There were suggestions to consider distinguishing between day and night however there was not a decision to harmonize further details. |
| Predominant breastfeeding | Infant receives breast milk as the predominant source of nourishment. Infant may also receive other liquids (water, fruit-juice, water-based drinks, ritual fluids) or vitamins, minerals or medicines.    Infant may NOT receive non-human milk or food-based fluids. | Adapted from WHO definition (20)    The group agreed that documentation of feeding practices beyond exclusive breastfeeding was needed. | Breast milk will be considered the “predominant source of nourishment” if the infant is only receiving non-milk liquids. Feedback from the group indicated it would be difficult to assess frequency of breast milk vs. other liquids to make a determination. |
| Partial breastfeeding | Infant receives breast milk and infant formula or non-human milk (also may receive any other liquids). | Added in meeting on May 26, 2020 to capture breastfeeding that includes some non-human milk (which is not part of the predominant breastfeeding definition) | Will require studies to capture formula and non-human milk separately from other liquids. |
| No breastfeeding | Infant receives no breast milk. | Added in meeting on May 26, 2020 to capture any infants who do not receive breastmilk. This is thought to be uncommon but is important in the context of studies providing maternal supplements. |  |
| Solid or semi-solid feeding | Infant receives solid, semi-solid, or soft foods. | Adapted from WHO definition (20)    Will allow combinations with other feeding variables to get “complementary feeding” with and without breast milk |  |
| Age at introduction of solid foods | Age at which caregivers begin to provide solid, semi-solid, or soft foods. | Will allow characterization of the age when foods are started, which can be compared to the recommendation to start at 6 months |  |
| Complementary feeding (6-<24 months) | Infant receives semi-solid foods (any food or liquid including non-human milk) in addition to breast milk. | Adapted from WHO definition (20)    This variable will be created from questions on breastfeeding and feeding solid foods | May decide to create another category/variable for non-breastfed children |
| Early initiation of breastfeeding | Infant was breastfed within 1 hour of birth (includes infants who die beyond one hour of birth). | Adapted from WHO definition (20) | Information on breastfeeding may not be available for infants that die soon after birth. |
| Exclusive breastfeeding duration | Total time that infant was exclusively breastfed (in weeks)  [could also assess breastfeeding duration past introduction of complementary food] | Continuous exclusive breastfeeding from birth (compiled/constructed from individual assessments) may yield different information compared to the age at introduction of solid foods |  |
| Minimum dietary diversity score (6-<24 months) | Children who receive foods from 5 or more out of 8 food groups:  -breastmilk  -grains, roots, tubers  -legumes and nuts  -dairy products (milk, yogurt, cheese)  -flesh foods (meat, fish, poultry, liver/organ meats)  -eggs  -vitamin A rich fruits and vegetables  -other fruits and vegetables  Consumption of any amount of food from each food group is sufficient to “count”, i.e., there is no minimum quantity except if an item is only used as a condiment | Adapted from WHO definition (20)    Included because studies will be continuing assessments past 6 months of age. The group agreed that assessment of feeding adequacy was needed, however plans for pooled analysis of infant growth currently go from birth to 6 months. |  |
| Minimum meal frequency (6 to < 24 months) | Children who receive solid, semi-solid, or soft foods (but also including milk feeds for non-breastfed children) the minimum number of times or more. Minimum is defined as:  -2 times for breastfed infants 6-8 months  -3 times for breastfed children 9-23 months  -4 times for non-breastfed children 6-23 months  “Meals” include both meals and snacks (other than trivial amounts), and frequency is based on caregiver report | Adapted from WHO definition (20)    Included because studies will be continuing assessments past 6 months of age. The group agreed that assessment of feeding adequacy was needed, however plans for pooled analysis of infant growth currently go from birth to 6 months. |  |
| MATERNAL DIETARY INTAKE | | | |
| Kcals per day (dietary intake) | Mean kcals consumed per day in maternal diet (not including BEP supplement) | Main purpose of assessing maternal diet is to examine substitution    Also will allow examination of underlying distributions of intake | Try to align dietary assessment with blood draws and food insecurity assessments    Try to align late pregnancy dietary assessment across studies (32-36 weeks?)    Will not harmonize dietary assessment tools |
| Carbohydrates per day (dietary intake) | Mean carbohydrate (g) consumed per day in maternal diet (not including BEP supplement) | Include quantification of all macronutrients | Micronutrients can be analyzed within individual studies but will not be part of harmonization |
| Protein per day (dietary intake) | Mean protein (g) consumed per day in maternal diet (not including BEP supplement) | Include quantification of all macronutrients |  |
| Fat per day (dietary intake) | Mean fat (g) consumed per day in maternal diet (not including BEP supplement) | Include quantification of all macronutrients |  |
| MATERNAL SUPPLEMENT ADHERENCE (COMPLIANCE) | | | |
| Adherence | Mean percent of assigned supplement that was consumed (out of total eligible and supplementation time)    Pregnancy and lactation adherence will be separated | It is important to compare adherence across studies, even though studies assess adherence in different ways (e.g., self-report, sachet count, direct observation). | Some studies are capturing partially consumed supplements. It was recommended that studies attempt to do so if possible. |
| Total time on supplement | Total time BEP supplement consumed during pregnancy (weeks) and/or lactation (weeks) | Studies begin supplementation at different time points from early to mid-pregnancy and it will be important to summarize the total time for comparison. |  |
| Kcals per day (from supplement) | Mean kcals consumed per day from supplement | Although BEP formulations are similar, this variable will allow quantification of the average kcals consumed in each study. | Will require information from BEP energy composition, adherence, and total time on supplement |
| Protein per day (from supplement) | Mean protein (g) consumed per day from supplement | Although BEP formulations are similar, this variable will allow quantification of the average amount of protein consumed in each study. | Will require information from BEP protein composition, adherence, and total time on supplement |

**References**

1. International Fetal and Newborn Growth Consortium. The International Fetal and Newborn Growth Standards for the 21st Century (INTERGROWTH-21st) Study Protocol [Internet]. 2008. Available from: www.intergrowth21.org.uk

2. World Health Organization. 2018 Global reference list of 100 core health indicators (plus health-related SDGs). Geneva: World Health Organization; 2018.

3. World Health Organization. WHO child growth standards: length/height-for-age, weight-for-age, weight-for-length, weight-for-height and body mass index-for-age: methods and development [Internet]. 2006 [cited 2021 Aug 11]. Available from: https://www.who.int/publications-detail-redirect/924154693X

4. Papageorghiou AT, Kennedy SH, Salomon LJ, Ohuma EO, Cheikh Ismail L, Barros FC, et al. International standards for early fetal size and pregnancy dating based on ultrasound measurement of crown-rump length in the first trimester of pregnancy. Ultrasound Obstet Gynecol. 2014 Dec;44(6):641–8.

5. Papageorghiou AT, Kemp B, Stones W, Ohuma EO, Kennedy SH, Purwar M, et al. Ultrasound-based gestational-age estimation in late pregnancy. Ultrasound Obstet Gynecol. 2016 Dec;48(6):719–26.

6. Stevens B, Buettner P, Watt K, Clough A, Brimblecombe J, Judd J. The effect of balanced protein energy supplementation in undernourished pregnant women and child physical growth in low- and middle-income countries: a systematic review and meta-analysis. Maternal & Child Nutrition. 2015;11(4):415–32.

7. Knight M, INOSS. The International Network of Obstetric Survey Systems (INOSS): benefits of multi-country studies of severe and uncommon maternal morbidities. Acta Obstet Gynecol Scand. 2014 Feb;93(2):127–31.

8. Stirnemann J, Villar J, Salomon LJ, Ohuma E, Ruyan P, Altman DG, et al. International estimated fetal weight standards of the INTERGROWTH-21st Project. Ultrasound Obstet Gynecol. 2017 Apr;49(4):478–86.

9. Villar J, Ismail LC, Victora CG, Ohuma EO, Bertino E, Altman DG, et al. International standards for newborn weight, length, and head circumference by gestational age and sex: the Newborn Cross-Sectional Study of the INTERGROWTH-21st Project. The Lancet. 2014 Sep 6;384(9946):857–68.

10. Physical status: the use and interpretation of anthropometry. Report of a WHO Expert Committee. World Health Organ Tech Rep Ser. 1995;854:1–452.

11. Xu H, Simonet F, Luo ZC. Optimal birth weight percentile cut-offs in defining small- or large-for-gestational-age. Acta Paediatr. 2010 Apr;99(4):550–5.

12. The DHS Program - Demographic and Health Surveys Program. Key Indicators Survey: Family Planning Questionnaire [Internet]. [cited 2021 Aug 11]. Available from: https://dhsprogram.com/methodology/Survey-Types/KIS.cfm

13. Institute of Medicine (US) and National Research Council (US) Committee to Reexamine IOM Pregnancy Weight Guidelines. Weight Gain During Pregnancy: Reexamining the Guidelines [Internet]. Rasmussen KM, Yaktine AL, editors. Washington (DC): National Academies Press (US); 2009 [cited 2021 Aug 11]. (The National Academies Collection: Reports funded by National Institutes of Health). Available from: http://www.ncbi.nlm.nih.gov/books/NBK32813/

14. ACOG Practice Bulletin No. 202: Gestational Hypertension and Preeclampsia. Obstet Gynecol. 2019 Jan;133(1):1.

15. WHO. Haemoglobin concentrations for the diagnosis of anemia and assessment of severity. Geneva: World Health Organizati; 2011.

16. Rohner F, Namaste SM, Larson LM, Addo OY, Mei Z, Suchdev PS, et al. Adjusting soluble transferrin receptor concentrations for inflammation: Biomarkers Reflecting Inflammation and Nutritional Determinants of Anemia (BRINDA) project. Am J Clin Nutr. 2017 Jul;106(Suppl 1):372S-382S.

17. World Health Organization. Preterm birth [Internet]. 2018 [cited 2021 Aug 11]. Available from: https://www.who.int/news-room/fact-sheets/detail/preterm-birth

18. Zupan J, Åhman E. Neonatal and perinatal mortality: country, regional and global estimates [Internet]. Geneva: World Health Organization; 2006. 69 p. Available from: https://apps.who.int/iris/handle/10665/43444

19. Imdad A, Bhutta ZA. Maternal nutrition and birth outcomes: effect of balanced protein-energy supplementation. Paediatric and Perinatal Epidemiology. 2012;26(s1):178–90.

20. World Health Organization. Indicators for assessing infant and young child feeding practices: definitions and measurement methods. 2021;
